# Large Optical Nonlinearity of the Activated Carbon Nanoparticles Prepared by Laser Ablation

**DOI:** 10.3390/nano11030737

**Published:** 2021-03-15

**Authors:** Yasin Orooji, Hamed Ghanbari Gol, Babak Jaleh, Mohammad Reza Rashidian Vaziri, Mahtab Eslamipanah

**Affiliations:** 1 College of Materials Science and Engineering, Nanjing Forestry University, Nanjing 210037, China; 2 Department of Physics, Faculty of Science, Bu-Ali Sina University, Hamedan 65174, Iran; hamed.gh053@gmail.com (H.G.G.); mahtabes740@gmail.com (M.E.); 3 Photonics and Quantum Technologies Research School, Nuclear Science and Technology Research Institute, Tehran P.O. Box 14399511-13, Iran

**Keywords:** activated carbon, carbon nanoparticles, laser ablation, nonlinear optical material

## Abstract

Carbon nanoparticles (CNPs) with high porosity and great optical features can be used as a luminescent material. One year later, the same group investigated the NLO properties CNPs and boron-doped CNPs by 532 nm and 1064 nm laser excitations to uncover the underlying physical mechanisms in their NLO response. Hence, a facile approach, laser ablation technique, was employed for carbon nanoparticles (CNPs) synthesis from suspended activated carbon (AC). Morphological properties of the prepared CNPs were studied by transmission electron microscopy (TEM) and scanning electron microscopy (SEM). UV-Vis and fluorescence (FL) spectra were used to optical properties investigation of CNPs. The size distribution of nanoparticles was evaluated using dynamic light scattering (DLS). The nonlinear optical (NLO) coefficients of the synthesized CNPs were determined by the Z-scan method. As a result, strong reverse saturable absorption and self-defocusing effects were observed at the excitation wavelength of 442 nm laser irradiation. These effects were ascribed to the presence of delocalized π-electrons in AC CNPs. To the best of our knowledge, this is the first study investigating the NLO properties of the AC CNPs.

## 1. Introduction

Carbon is one of the most standout elements on the earth and can be formed in different forms such as carbon nanoparticles, carbon nanotube, and graphene [1,2]. Recently, carbon-based nanomaterials, including carbon nanotubes (CNTs), CNPs, and graphene, have been noticed due to their superior properties like high porosity, high adsorption capacity, nontoxicity, adjustable morphology, and simple preparation [3,4]. CNPs with small size, good biocompatibility and chemical stability, high hydrophilicity, and great flexibility show interesting optical properties known as luminescent materials [5,6]. Their great conductivity and electrochemical activity and high surface area have caused to application of them in energy devices like batteries and supercapacitors [7,8,9,10]. It is worth mentioning that CNPs are more interested than other carbon nanostructures due to their facile and low-cost synthetic approaches containing physical and chemical methods. Thermal/hydrothermal [11], electrochemical synthesis [12], and acidic oxidation [13] are some CNPs chemical synthesis methods. Since chemical methods may be damaging to environmental, physical methods such as plasma treatment [14] and laser ablation [15] can be used as a suitable alternative for chemical methods.

Laser ablation in liquid (LAL) media is one of the simplest ecofriendly methods for CNPs synthesis [15]. In the LAL process, size and morphology of the produced NPs depend on both the laser parameters (wavelength, frequency, power, etc.) and the used liquid medium [16]. This promising method has a number of advantages such as decreasing the effect of heat on the target, lowering the synthesis time, and using clean environments without any chemical pollutions [17,18]. Furthermore, the synthesized NPs disperse in the liquid media and produce colloidal suspensions during the LAL process, which can simplify the utilization of the produced NPs for in vivo applications [17,19,20]. Some researchers have reported the synthesis of CNPs using the LAL process of suspended particles in liquid as targets. For example, Hu et al. synthesized luminescent CNPs by laser ablation of dispersed carbon black in poly(ethylene glycol) solution [21].

Moreover, Małolepszy et al. used the laser ablation process for the synthesis of fluorescent CNPs from suspended reduced graphene oxide in deionized water, isopropyl alcohol (IPA), acetone, and PEG200 liquids [22]. Briefly speaking, there are three steps in the LAL process of suspended particles in liquid as targets: first, when the pulsed laser is focused and the radiation is started, it penetrates into the suspended particles [23]. In the second step, the laser-induced melting-evaporation occurs for the large particles and produces atoms and molecules. Afterwards, atoms and molecules aggregate and form nanostructures with different shapes and sizes [24]. In the third step, the thermal ejection of electrons occurs from the nanostructure surface that leads to the formation of positive charges which has as a result the laser-induced Coulomb explosion. Electrostatic repulsion between different parts of the primary particles arises and causes the production of finer particles due to the crushing of the larger ones [24]. AC is an ideal family of carbon materials with porosity spaces which are surrounded with carbon atoms and has special properties such as high surface area, large pore volumes, good thermostability at high temperatures, and small pore sizes (<1 nm) properties [25,26,27]. Because of its unique properties, AC can be used to CNPs preparation.

In semiconducting or insulating materials, single-photon linear absorption may occur if the incident photon energy be of the bandgap energy order. This is a constant effect independent of the incident light intensity. When this single-photon process becomes intensity-dependent, two different effects of saturable absorption or reverse-saturable absorption can occur. In the case of a two-level material, larger or smaller absorption of the excited state concerning the ground state leads to reverse-saturable or saturable absorption effects, respectively. In a saturable absorber, the absorption coefficient decreases with increasing the light intensity; while in a reverse-saturable absorber, the situation is exactly reverse because of the ground state depletion. At high-intensity optical fields, there is also the possibility of bandgap bridging by synchronous absorption of more than one photon, with the condition that sum of the photon energies exceeds the energy gap. These kinds of NLO processes are called multiphoton absorption.

In the simplest case, just two photons are involved, and the process is known as two-photon absorption. Materials with different dominant NLO processes have attracted a lot of interest. For instance, saturable absorbers are used for Q-switching of high-power lasers [28]. Reverse-saturable absorbers and materials with two- and multi-photon absorptions are also used as optical limiters and in nonlinear microscopy, 3D imaging, and nonlinear spectroscopy [29]. In order to design and develop more efficient NLO devices, great attention has turned to find materials with larger NLO response in recent years [30,31,32].

In quest of new nonlinear optical (NLO) materials besides graphenes, fullerenes, and carbon nanotubes, little is known about the NLO properties of CNPs [33]. Recently, it was noticed that CNPs possess comparable NLO properties to other types of NLO materials, such as perovskites and antimonenes [34]. They can be easily and inexpensively synthesized and, hence, are often regarded as favorable materials for NLO devices. Furthermore, they have abundant delocalized-electrons that ensure their good NLO performance and make them appropriate candidates in such applications [35]. In 2014, it was noticed that CNPs exhibit negative nonlinear refractivity using a 532 nm laser source by the Z-scan method [33]. One year later, the same group investigated the NLO properties CNPs and boron-doped CNPs by 532 nm and 1064 nm laser excitations to uncover the underlying physical mechanisms in their NLO response [36]. Following these two pioneering works, subsequent studies in many other groups, especially in recent years, have focused on investigating the NLO properties of CNPs and CNPs doped with other materials [34,35,37,38,39,40,41]. However, to the best of our knowledge, there is no study on investigating the NLO properties of the AC CNPs, and much work is required in this area.

Table 1 summarizes the other reports based on the used synthesis method of CNPs, their size range, and the corresponding possible applications or interesting optical properties.

Herein, CNPs were prepared by LAL on the suspended AC in heptane medium. Structural, optical, and NLO properties of the prepared CNPs were studied. Employing the Z-scan method, it is shown that CNPs exhibit large optical nonlinearities by excitation with 442 nm laser radiation.

## 2. Materials and Methods

### 2.1. Materials and Instruments

Commercial granular AC (Merck, Germany) with a mean diameter of 1.5 mm andspecific surface area of 961.1 m^2^/g was utilized in this work [55]. A fiber laser (RFL-P30Q, China) with the wavelength of 1064 nm, maximum power of 30 W, and frequency of 20 kHz was used for the LAL process. To obtain homogenous suspension of AC and n-heptane, the ultrasonic bath (DSA100-SK2-4.0L, China) with voltage of 220 V, power of 100 W, and frequency of 40 kHz was utilized. The fabricated colloidal samples’ optical properties were perused using ultraviolet-visible spectroscopy (UV-Vis, JASCO V-630, Japan) and fluorescence spectroscopy (Perkin Elmer LS50B, United Kingdom) techniques. DLS (Zetasize Nano ZS, Malvern) technique was used to determine the size distribution profile of CNPs. SEM (TESCAN MIRA3-XMU, Brno-Kohoutovice, Czech Republic) and TEM (CM120, Netherlands) images were used for morphological investigation of nanoparticles. Energy-dispersive X-ray spectroscopy (EDX) assay was used to determine the presence of elements in CNPs. NLO coefficients of the fabricated samples were measured by the Z-scan method.

### 2.2. Laser Ablation of Suspended AC

**Figure 1:**
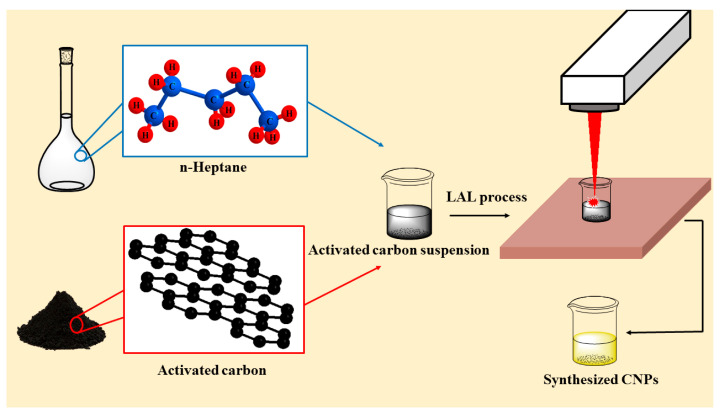
Schematic representation of the adopted experimental procedure for preparation of CNPs.

The laser ablation was carried out in n-heptane medium. To this aim, 5 mg of AC was dispersed in 10 mL of n-heptane solvent using 20 min ultrasonication. Afterwards, the dispersion was exposed to laser irradiation for 130 min. The color of suspension was changed to yellowish during the laser irradiation, which confirmed the CNPs formation. Finally, the irradiated sample was filtered and dried. Figure 1 illustrates the schematic representation of the synthesis process.

### 2.3. Z-Scan Measurements

**Figure 2:**
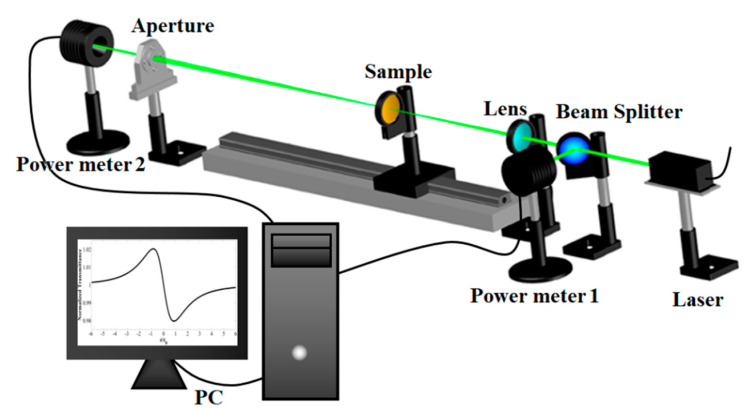
Scheme of the employed Z-scan system for measuring the nonlinear optical (NLO) properties.

The well-known Z-scan method was used for measuring the NLO coefficients. Z-scan is a rapid, easy, and sensitive method for measuring the NLO coefficients of materials [56,57]. This method has attracted much interest and is being extensively used to determine the NLO coefficients of materials [58]. Figure 2 depicts the scheme of the used experimental Z-scan setup in this study. The beam splitter divides the emitted laser beam (CW He-Cd, 442 nm, 150 mW). The first power meter (power meter 1) measures the power of the first half as the reference power. A converging lens (f = 25 cm) focuses the other half of the laser beam (spot size ~20 µm at focus). This divided part of the laser beam passes respectively through the sample and the aperture and reaches the second power meter (power meter 2), which measures its power as the signal power. The diameter of the used aperture was 1.5 mm in the acceptable range for diameter of apertures in Z-scan measurements [59]. The one-dimensional translation stage moves the position of the sample along the optical axis (the Z-axis). The normalized transmittance T was continuously measured by calculating the ratio of signal to reference beam powers. The Z-scan measurement system components were placed on a firm optical bench to reduce the adverse vibration noise effects. The measurements were also made in the dark environment to minimize the noise from the interference of extraneous light.

Theoretically, at high-intensity fields near the focal point of the converging lens, the sample behaves like a feeble lens whose focal length is proportional to the nonlinear refractive index *n_2_* of the sample and the position along the Z axis [60]. In the so-called closed-aperture Z-scan, the presence of the far-field aperture enables the setup to analyze the tiny distortions induced by this weak *n_2_*–dependent lens in the signal beam [61]. By lateral displacement of the aperture from the beam propagation axis, the open-aperture configuration, the nonlinear absorption coefficient will be measured by recording the entire signal beam. Without the aperture, the induced tiny distortions by the feeble *n_2_*–dependent lens are insignificant and the changes of the signal beam power are only due to the NLO absorption of the sample [62]. The required curve equations for fitting on the normalized transmittance data and finding the NLO constants [63] can be theoretically obtained by describing the propagation of laser beam in the lens-like media by a suitable model [64].

## 3. Results and Discussion

**Figure 3:**
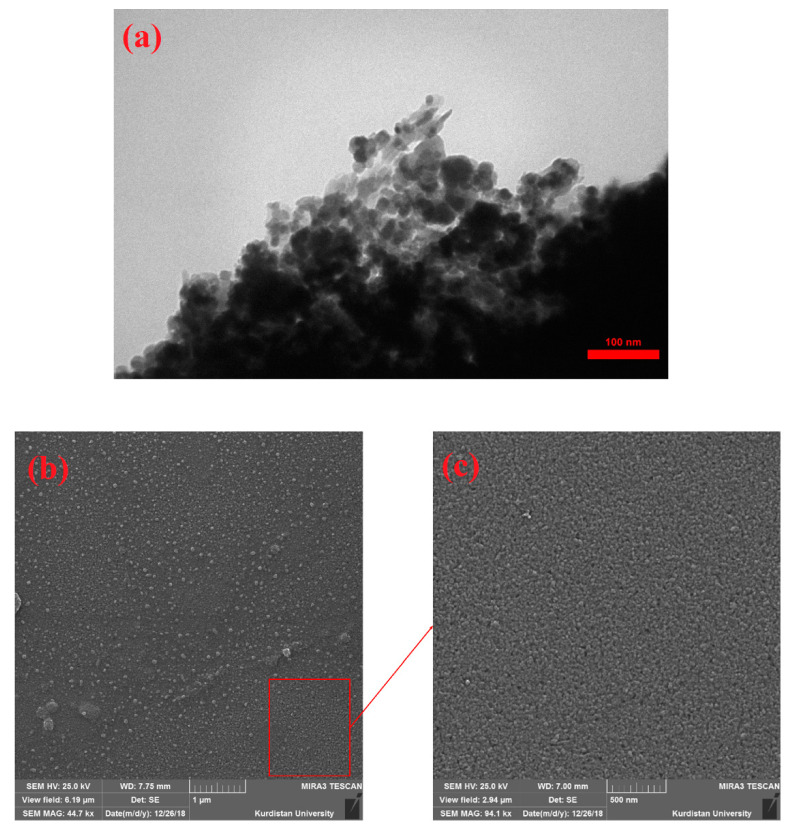
(**a**) TEM and (**b**,**c**) SEM images.

**Figure 4:**
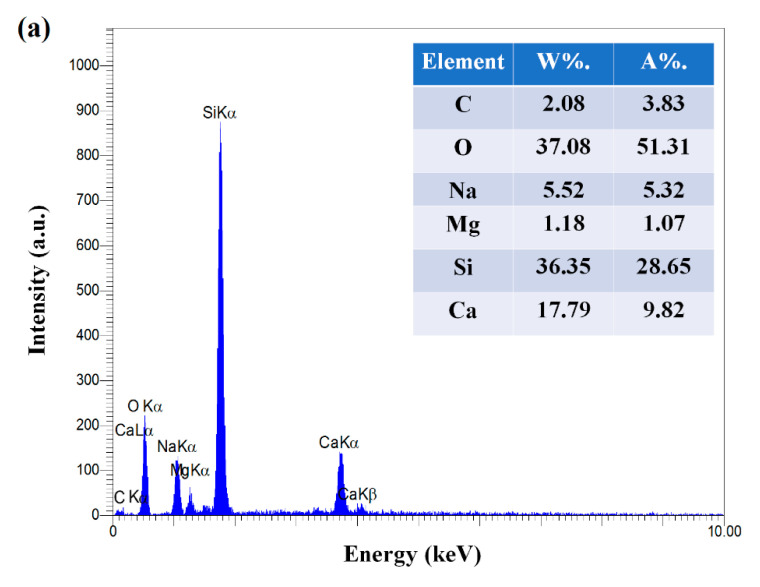

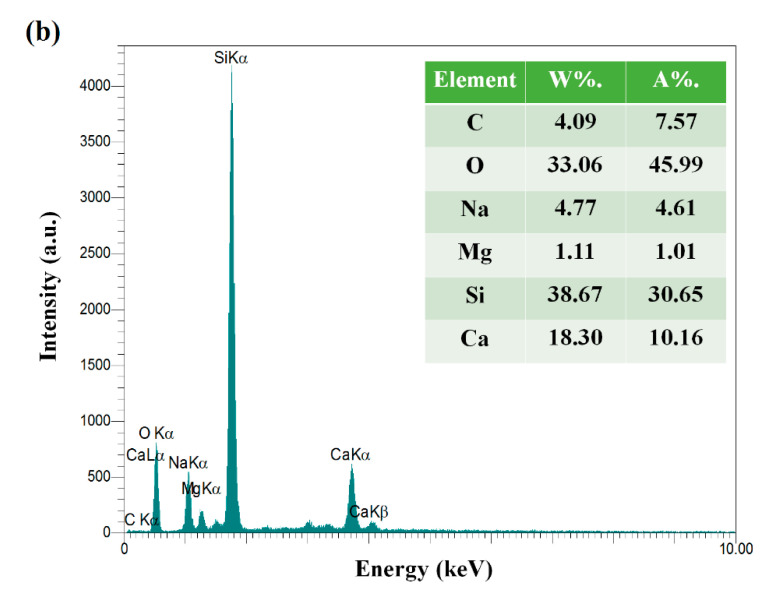
Energy-dispersive X-ray spectroscopy (EDX) spectra of (**a**) laboratory slide and (**b**) CNPs.

For morphological characterization of the fabricated CNPs by SEM and TEM analyses, NPs colloidal solution was dried on laboratory slides. As shown in Figure 3a–c, CNPs have been successfully formed in irregular spherical shapes due to the LAL process. Furthermore, the EDX spectra were utilized to analyze the presence of elements (Figure 4). As shown in Figure 4a,b, the synthesized sample has more amount of C element compared with laboratory slide.

**Figure 5:**
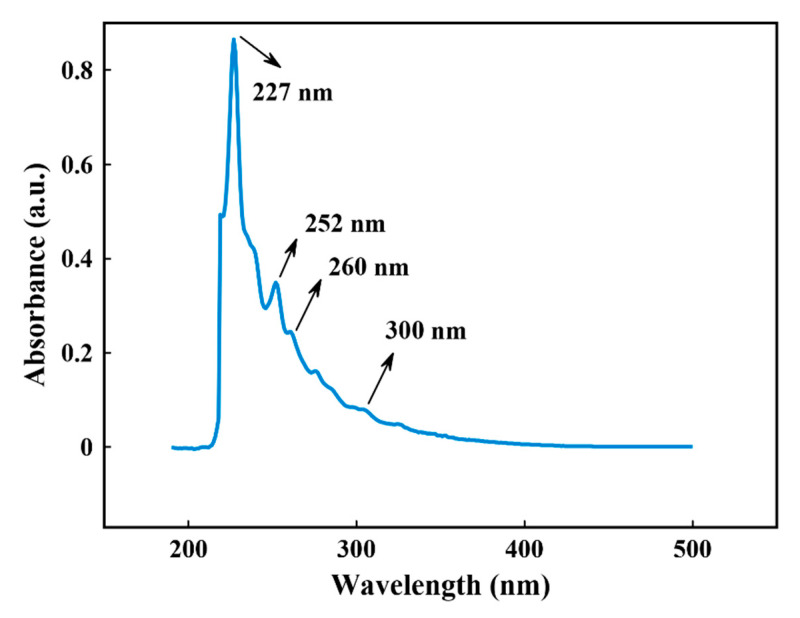
UV-Vis absorption spectrum of the CNPs suspension.

UV-Vis and fluorescence (FL) spectra were recorded to study the linear optical properties of the synthesized CNPs. Figure 5 demonstrates the UV-Vis spectrum of the CNPs suspension. Generally, CNPs have absorption peaks in the range of 180–280 nm [5]. According to Figure 5, three absorption peaks appear at 227 nm, 252 nm, and 260 nm, which are related to the π-π* transition of C=C bonds in CNPs [43]. The weak absorption peaks at around 300 nm appear probably due to n-heptane molecules or their decomposition [12]. In addition, the obtained UV-Vis spectra of CNPs has more number of absorption peak compared with UV-Vis spectra of the prepared CNPs by LAL method in water [65,66].

**Figure 6:**
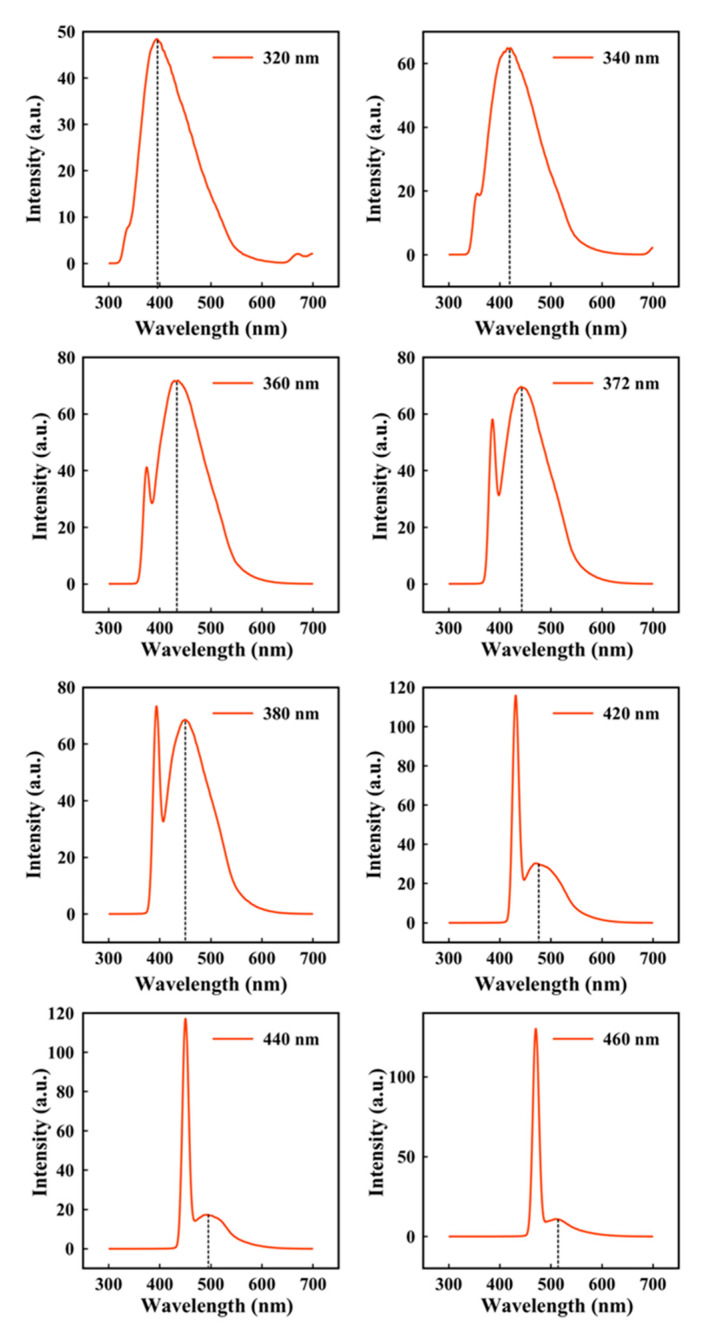
Fluorescence emission spectra of the prepared CNPs at various excitation wavelength.

The CNPs FL emission spectra at different excitation wavelengths were recorded. The results are shown in Figure 6 and listed in Table 2. It is clear that the maximum FL emission of CNPs is at 436 nm by exciting the sample at the wavelength of 360 nm. Moreover, a red shift can be discerned in the maximum FL emission of the sample by increasing the excitation wavelength. The peak intensity also decreases by increasing the excitation wavelength. Therefore, the FL emission of CNPs depends on the excitation wavelength, which is known to be due to the inherent size effect of CNPs [67].

**Figure 7:**
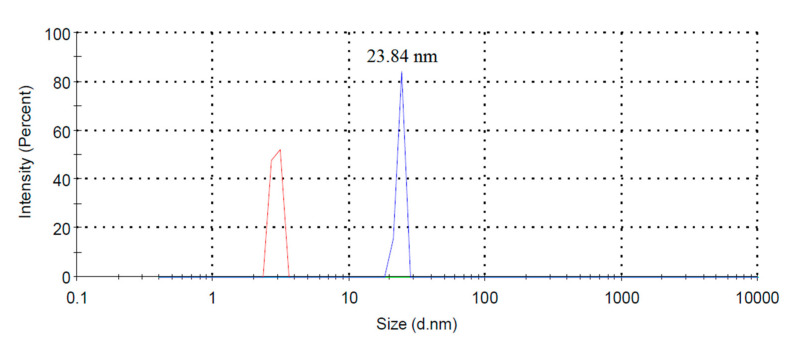
Size distribution of the synthesized CNPs.

DLS measurements determined the size and size distribution of the fabricated CNPs. As shown in Figure 7, the size of suspended CNPs in n-heptane is in the range of 2–4 nm and 18–28 nm, and their average size is 23.84 nm.

**Figure 8:**
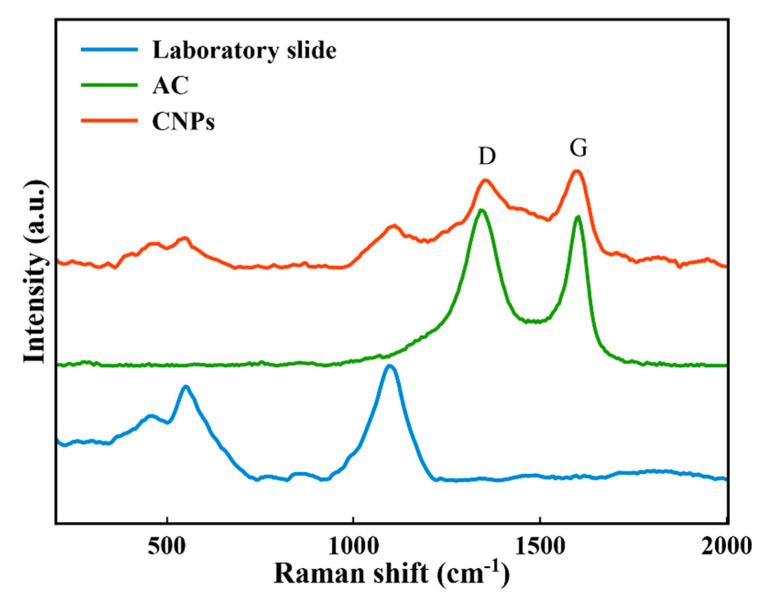
Raman spectra of the laboratory slide, activated carbon (AC), and the fabricated CNPs.

To study the structural properties, Raman characterization of the laboratory slide, AC, and the fabricated sample were performed at room temperature. The obtained results are shown in Figure 8. As can be verified, two peaks at approximately 1340 cm^−1^ and 1600 cm^−1^ are observable for AC spectrum, which are related to the D (the diamond) and the G (the graphitic) bands, respectively [68]. The D band of CNPs appeared at about 1350 cm^−1^, and the intensity of both D and G bands was reduced after laser irradiation. The D and the G bands have appeared due to the Sp^3^-bonded structure and the Sp^2^ bonds vibrations, respectively [68,69]. Compared with the synthesized CNPs in water, the intensity difference between D and G bands is lower [69], suggesting that the decomposition of n-heptane as an organic solvent may be enhanced which lead to carbon species fabrication [70].

**Figure 9:**
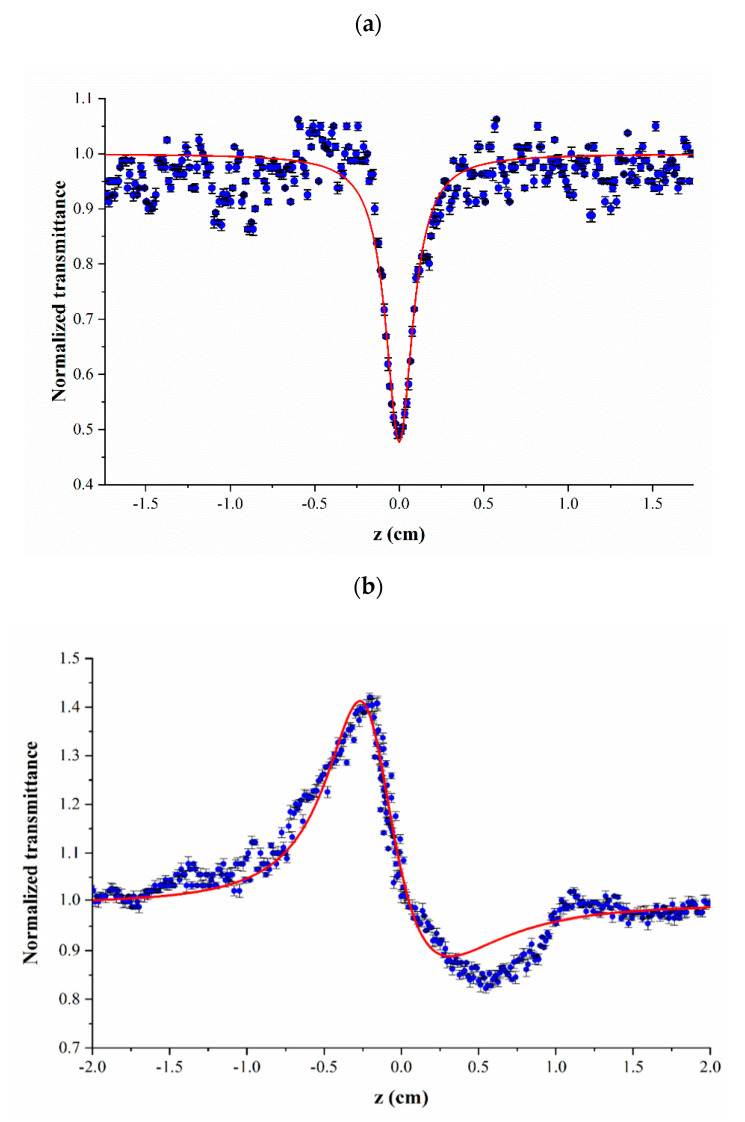
(**a**) open- and (**b**) closed-aperture Z-scan data points of the synthesized CNPs associated with their fitting curves.

The Z-scan technique determined NLO coefficients of the CNPs. Figure 9 shows the results of open- and closed-aperture Z-scan measurements. The next equation was used for fitting the open-aperture data and finding the value of β [71,72]:

(1) $$T_{\mathrm{open}}\left(z\right)=\frac{\ln\left(1+q_{0}\right)}{q_{0}}$$

With

(2) $$q_{0}=\frac{\beta I_{0}L_{\mathrm{eff}}}{1+x^{2}}$$

where $x=z/z_{0}$ and $L_{\mathrm{eff}}=\;\left(1-\exp\left(-\alpha_{0}L\right)\right)/\alpha_{0}$ is the effective thickness, with α_0_ and L being the linear absorption coefficient and the sample real thickness, respectively. $z_{0}$ and $I_{0}$ are also the Rayleigh length and the on-axis intensity at the focus of the converging lens.

Visual inspection of Figure 9b indicates the asymmetric shape of the Z-scan curve, which implies the large NLO response of the prepared CNPs. When this is the case, the general Z-scan theory based on the small phase shift approximation [57] cannot well fit the measured data, and an extended Z-scan theory should be used instead [72]. Therefore, the next equation was applied as the fitting function for determining the value of n_2_ [72]:

(3) $$T_{\mathrm{closed}}\left(z\right)=\frac{1}{1-\frac{\left(4x-\eta \right)}{{\left(1+x^{2}\right)}^{2}\left(1+q_{0}\right)}\Delta \Phi_{0}+\frac{\left(4+\eta^{2}\right)}{{\left(1+x^{2}\right)}^{3}{\left(1+q_{0}\right)}^{2}}\Delta \Phi_{0}{}^{2}}$$

where $\eta =\beta /\left(2kn_{2}\right)$ and $\Delta \Phi_{0}=kn_{2}I_{0}L_{\mathrm{eff}}$. Hence, after extracting the value of β by fitting Equation (1) on the open-aperture data, the value of n_2_ was obtained using Equation (3) as the fitting function for closed-aperture results Z-scan measurements. Nonlinear least-squares analysis was implemented in MATLAB for fitting these two equations on the z-scan experimental data. The extracted values of β and n_2_ for the synthesized CNPs are listed in Table 3.

The positive value of β is compatible with the observable valley in the open-aperture signal in Figure 9a and indicates that reverse saturable absorption is the dominant nonlinear absorption mechanism in CNPs, which is consistent with a number of previous reports [33,34,35,38,40,73]. The observed strong reverse saturable absorption response of the synthesized AC CNPs in this work elucidates how they can be promising candidates for optical limiting applications. The negative value of the nonlinear refractive index in Table 2 is also compatible with the discernible peak-valley configuration (i.e., a pre-focal transmission maximum followed by the post-focal transmission minimum) in Figure 9b, which is also consistent with some previous studies [33,40].

The presence of π-electrons that can delocalize within the backbone of the organic compounds is considered the major reason for observation of NLO properties in these materials [74,75]. NLO properties that originate from π-electrons of sp^2^ hybridized carbon in graphene and its derivatives have also attracted great interest [76,77]. Regarding the CNPs, it is recently shown that their NLO properties are caused by induction of higher absorption cross sections of the excited-states by π-conjugated structure [35] and the ratio of sp^2^ and sp^3^ bonded carbon atoms [33]. As it is indicated before, the D and the G bands in Figure 8 are due to the Sp^3^ and the Sp^2^ bond vibrations, respectively. As can be verified in this figure, the Sp^2^ carbon atoms are dominant in the laser fabricated AC CNPs, explaining the large optical nonlinearities observed in this material.

## 4. Conclusions

Due to their favorable characteristics, CNPs are now widely used in various fields such as biological imaging, drug delivery, fluorescent sensor design, multicolor LED production, energy conversion, and storage, etc. [78]. Indeed, the low cost and synthesis facility of CNPs have made them promising nanomaterials for optoelectronic and photonic applications. In this paper, for the first time to our knowledge, it is shown that the synthesized AC CNPs exhibit large NLO refractivity and absorption by excitation with 442 nm laser radiation. The present results indicate that AC CNPs are also very promising candidates for designing NLO devices and for various other photonic applications. We expect this first study on the NLO properties of the AC CNPs to foster several other groups investigating the NLO properties of AC CNPs with other laser excitation characteristics. Uncovering the underlying physical mechanisms in the NLO response with more details and functionalization of the AC CNPs in NLO devices are the other directions that can be followed up in future studies.

## Figures and Tables

**Table 1:** Comparison of the synthesized carbon nanoparticles (CNPs) size by different methods.

Nanostructure	CNPs Synthesis Method	CNPs Size (nm)	Properties and Application	Ref.
Carbon nanoparticles	LAL	4–20	-Good photoluminescence -Can be used for bioimaging	[42]
Nitrogen-doped carbon nanodots (N-CND) and Starch Derived Carbon Nanodots (C-CND)	microwave-assisted hydrothermal precursor carbonization	2.0 ± 0.24 (C-CND) and 2.4 ± 0.25 (N-CND)	-High photoluminescence quantum yield -Long-term stability -Having stable emission	[43]
Boron-doped carbon dots	Microwave heating	2–6	-Robust blue fluorescence under UV excitation -Large nonlinear optical	[36]
Nitrogen-doped carbon nanoparticles	Microwave oven	5.5 ± 1.5	-Highly fluorescent	[44]
Carbon nanoparticles	Thermal pyrolysis	20–50	-Highly fluorescent -Excellent photoluminescent -Used as a metal sensing probe	[45]
Carbon nanoparticles	Thermal carbonization	-	-Synthesized nanoscale particle size -Used as a supercapacitor -Highly specific capacitance and excellent long-term cycle stability	[9]
Carbon nanoparticles	Stirring and reflux method	115	- Improved release of the drugs	[46]
Carbon nanoparticles	Dehydration of hyaluronic acid and carbonized hyaluronic acid	<20	-Flexibility -Biocompatible and low cytotoxicity -Used for in vitro and in vivo bioimaging	[47]
Carbon Nanoparticles	Hydrothermal carbonization and high-temperature annealing	120	-Used as an anode for lithium-ion Battery	[48]
Carbon Nanoparticles	Thermally-assisted carbonization	-	-synthesized nanoparticles in small size -Strong blue luminescence -Used for sensing of metal ions	[49]
Carbon dots	Microwave oven	<10	-Highly biocompatible -Great fluorescent property -Used for cell imaging	[50]
Carbon Nanoparticles	acid treatment of naturally occurring d-glucose followed by heating	<5	-Used for sensing of metal ions -Used for in vivo imaging	[51]
Carbon Nanoparticles	Hydrothermal treatment	20–40	- Highly photoluminescent and photo-stability -Low toxicity and good biocompatibility -Used for in vitro and in vivo imaging	[52]
Carbon Nanoparticles	Light-induced process	40	-Used for sensing of metal ions -Used as a photocatalyst for hydrogen evolution	[53]
Nitrogen-doped carbon quantum dots	Microwave-assisted	2.47 ± 0.84	-Size and surface controllable of synthesized NPs -Fluorescent emission -Excellent solubility in water	[54]
Carbon nanoparticles	Laser ablation in n-heptane	23.84	-Large optical nonlinearities with 442 nm laser radiation	This work

**Table 2:** The fluorescence wavelength at different excitation wavelength.

Excitation Wavelength (nm)	Fluorescence Wavelength (nm)
320	395
340	421
360	435
372	442
380	449
420	477
440	494
460	514

**Table 3:** The NLO constants n_2_ and *β* of the synthesized CNPs.

n_2_ (cm^2^/W)	*β* (cm/W)
−1.15 ± 0.09 × 10^−9^	1.49 ± 0.11 × 10^−4^

## Data Availability

Not available data.
